# Organizational Support for Nurses' Career Planning and Development: A Scoping Review

**DOI:** 10.1155/2024/8296762

**Published:** 2024-04-26

**Authors:** Hanna Kallio, Hanna Liljeroos, Marita Koivunen, Anne Kuusisto, Marja Hult, Mari Kangasniemi

**Affiliations:** ^1^University of Turku, Faculty of Medicine, Department of Nursing Science, Kiinamyllynkatu 10, Turku 20014, Finland; ^2^The Wellbeing Services County of Satakunta, Sairaalantie 3, Pori 28500, Finland; ^3^The Wellbeing Services County of Satakunta, Research and Development Unit, Sairaalantie 3, Pori 28500, Finland; ^4^South-Eastern Finland University of Applied Sciences, Patteristonkatu 3, Mikkeli 50100, Finland

## Abstract

**Aim:**

To systematically map and identify key knowledge on organizational support for nurses' career planning and development.

**Design:**

Scoping review.

**Methods:**

Systematic electronic searches were carried out with the CINAHL, PubMed, Scopus, and Web of Science databases in May 2022. The searches were limited to scientific, peer-review papers that were published in English from January 2012 to May 2022. Data were extracted and synthetized and are presented in tables and text. The review was conducted following the Preferred Reporting Items for Systematic Reviews and Meta-Analyses guidelines.

**Results:**

We identified 1,400 papers and 28 met the inclusion criteria. Organizations recognized nurses' career planning and development in relation to the individual's professional development and the organization's need to promote high-quality services and workforce engagement. The organizational support included strategic work to ensure there were adequate resources and purposeful vacancies and a structured framework based on objective qualification criteria and equal assessment. Organizations focused on sharing knowledge, structured career planning, and interpersonal support. Support within the nursing profession and multilayered interprofessional collaboration were also important.

**Conclusion:**

Nurses' career planning and development was linked to their personal development and the organization's aims and required support from both fellow nurses and other professionals. *Implications for the Nursing Management*. Identifying the organizational structures and methods that are needed to support nurses' career planning and development can help nursing management to evaluate and develop strategies that improve the attractiveness of a nursing career and nurses' engagement.

## 1. Introduction

The growing global shortage of healthcare workers highlights both the need to promote nursing as an attractive career [1, 2] and the importance of engaging the nursing workforce [3]. Previous research has addressed nurses' desire for professional and career development [1, 4, 5]. Nursing careers are typically directed towards leadership, academic [6], or educational work [7] but previous knowledge on career development opportunities in bedside nursing is limited [1, 4]. Poor career prospects [4, 6] and a lack of recognition [8] have decreased meaningfulness and satisfaction at nurses' work resulting in staff turnover [9, 10].

Hence, career planning and development (CP&D) is a topical issue for nursing workforce management [4, 6]. CP&D refers to a nurse's expectations and prospects for work goals, advancement, and progress in their career development [10, 11] based on continuing learning [10] and recognized expertise [4, 12]. Nurses' CP&D has been studied in relation to individual factors, namely, the inner motivation to select nursing as a career and developing their own excellence and progress in that career [1, 13]. The need for nurse's autonomy [8, 14] and personal resources [15], own responsibility, and motivation for CP&D has been highlighted [16]. However, research has also identified nurses' unfamiliarity with CP&D [17] as well as their neglect for building relationships to advance their careers [10].

An important factor for nurses' CP&D is organizational support and organizations' role has been emphasized in promoting CP&D to ensure nurses' efficiency, engagement [18], and care quality [5, 10]. Based on previous research, health and social care organizations who are committed to their staff tend to support nurses' CP&D with systematic, long-term strategic management and structures [19, 20]. The role of organizations and managerial engagement [21] has also been emphasized in relation to ensuring informed career guidance for nurses as well as the return of CP&D to the benefit of the organizations [17].

Previous reviews on career development in nursing have covered nurses' career intentions [8, 14], professional development [22, 23], career success [15], career path options [12], and the implementation of clinical ladder programs [21]. Instead, knowledge on organizational support for nurses' CP&D has been fragmented and needs to be brought together to provide the basis for the evidence-based development of nurses' working lives. It has been unclear what are the means for promoting nurses' CP&D, and identifying them is needed for the coherent development and evaluation of CP&D in organizations. It is essential that versatile means are available to support employees, which contributes to the equal treatment of them and, above all, their individual career plans. The study is also needed to identify the knowledge gaps that need to be addressed by further research. Thus, the aim of this scoping review was to systematically map and identify key knowledge on organizational support for nurses' CP&D. The research questions were as follows:

1. How has nurses' CP&D been defined by organizations?
2. How do organizations support nurses' CP&D in general?
3. What methods do organizations use to support nurses' CP&D?

## 2. Methods

### 2.1. Study Design

We conducted a scoping review on studies that focused on organizational support for nurses' CP&D. The scoping approach was chosen, as it was suitable for mapping and summarising previous fragmented research evidence on the topic and it was able to identify key concepts and research gaps [24]. The review was carried out in the following five stages [24]: (i) formulate the research questions, (ii) search for the relevant studies, (iii) select the studies to include in the review, (iv) chart the selected studies, and (v) report the results. The review was reported following the Preferred Reporting Items for Systematic reviews and Meta-Analyses extension for Scoping Reviews (PRISMA-ScR Checklist ([25], Supplementary file 1).

### 2.2. Search Methods and Selection and Evaluation of Studies

We identified the research questions based on preliminary literature searches and the PICO-structure where the population (P) was nurses, the intervention (I) was organizational means for CP&D, and the outcome (O) was career CP&D. Comparison or control (C) element was not suitable for his study. We carried out the electronic searches using the CINAHL, PubMed, Scopus, and Web of Science databases, which were the most relevant for the study topic. Following preliminary searches, we worked with an informatician to develop explorative search phrases that consisted of free words, fixed terms, and their combinations and synonyms for the two key fields of interests, nursing and career (Supplementary file 2). We limited the searches to scientific, peer-reviewed papers published in English from January 2012 to May 2022. The papers were included if they focused on nurses and organizations who supported their CP&D. The papers were independently selected by two researchers (HK and HL) who reviewed the titles and abstracts and the full texts. The eligible studies were included in the review.

The quality of studies was assessed to increase the transparency of the data [25]. Quality of empirical qualitative [26] and quantitative [27] and theoretical [28] studies was assessed according to the Joanna Briggs Institute's checklists and multimethod studies according to the criteria presented by Harrison et al. [29]. Evaluation of qualitative studies focused on ten criteria, quantitative studies on eight, and theoretical studies on six criteria. Each criterion received one or zero points. There were 13 evaluation criteria for multimethod studies each of which received 0–3 points (Supplementary file 3).

### 2.3. Data Analysis

We analysed the data by following the principles of inductive content analysis [30]. First, we tabulated the papers according to the year, location, aim, methods, scope of the nursing career, and main results (Tables 1 and 2). Then, we extracted the sentences and paragraphs that described nurses' CP&D and how patient care organizations supported it. The extracted material was then organized into groups based on the similarities and differences and named inductively based on their content. To abstract the data, we then brought the groupings together to form eight subcategories and three main categories (Table 3). Two researchers (HK and HL) carried out the analysis until the extraction phase. Then, it was finalized in collaboration with the entire research team. The analysis was conducted using NVivo v.12 software.

## 3. Results

### 3.1. Study Characteristics

**Figure 1:**
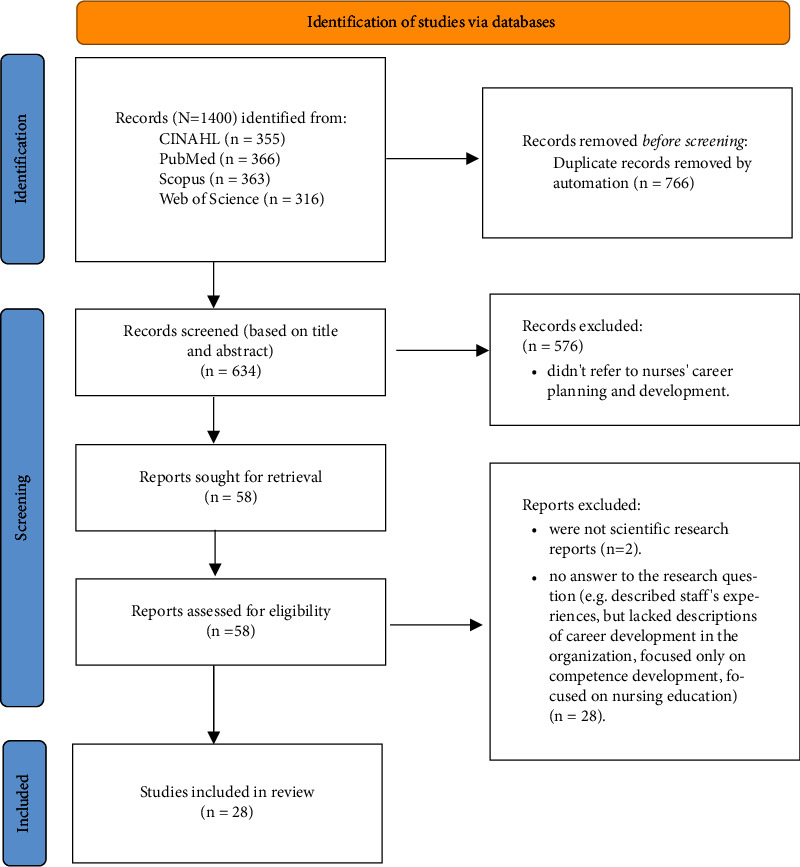
PRISMA flowchart.

The searches yielded 1,400 results; 58 full texts were read and 28 met the eligibility criteria and were included in the review as data (Figure 1). Of the selected papers, 14 were empirical studies; one used quantitative methods, ten used qualitative methods, and three used multiple methods. The other 14 were theoretical papers; seven were nonsystematic reviews, six were project reports, and one was a discussion paper. All the papers were published between 2012 and 2022. Eleven of the studies were conducted in Europe, nine in North America, five in Asia, and three in Australia (Tables 1 and 2).

The overall quality of papers was moderate (Supplementary file 3). In 10 qualitative studies, the scoring by paper varied between 3/10 and 9/10 (6.9 points out of 10 on average). Scrutinised by the evaluation criteria, the highest points in them were achieved by the congruity between the research methodology and the interpretation of results and data-based conclusions (10 out of 10 studies). Lowest points were achieved by the description of philosophical starting points and influence of researchers (3 out of 10 studies). One quantitative study included in the data received an overall score of 4/8. The three multimethod studies achieved 10, 17, and 19 points out of 39. In them, research aims was most clearly reported (9 out of 9 points) and methodological justifications, sampling, recruitment, and analysis less clearly reported (0 out of 9 points). Theoretical papers achieved scoring from 4 to 6 out of 6 points. In them, expertise basedness was unclear in some papers. For both empirical and theoretical studies, the evaluation criteria were not fully applicable (Supplementary file 3).

### 3.2. Definitions and Rationale for Nurses' CP&D

The nurses' CP&D in an organization were defined and justified from individual and organizational points of views. These contents were interlinked and created a basis for CP&D (Table 3).

#### 3.2.1. Individual Level: Increase in Personal Competency to Respond for New Work Challenges in the Organization

On an individual level, nurses' career planning was described as an intentional act that focused on their competencies and the kind of work that would support their career in the future [33]. Career development was described as an increase in the individual development of personal qualities [34], competencies [16], strengths and talents [35], role expansion, and professional development [36]. Challenges were increased [36, 37] and so was the authority that nurses needed to execute and prioritize new duties [37, 38] and the abilities they needed to deliver quality patient care [32]. The work needed to be challenging enough to drive career development [39, 40] and enable the individual to show creativity [34]. New or expanded roles needed to be documented in job descriptions [36] and job titles [37]. Advancing to higher positions resulted in larger salaries [36, 37]. Career development aimed to strengthen nurses' professional identity [41] and pride [38]. Nurses were respected by patients and the working community [37] and their power and authority in organizations [34], and their participation in organizational decision-making, had increased [41].

#### 3.2.2. Organizational Interest: Resources for Improving the Quality of Care and Services

Nurses' CP&D reflected an organization's commitment to staff development [31] and was connected to successful recruitment [41] and retention [40]. Organizations that supported nurses' careers showed that they recognized and valued their staff's skills and experiences [39] and their readiness to develop nursing career paths, including their attitudes, efforts, efficacy, and commitment [16]. From an organizational point of view, CP&D improved their performance, motivation, and productivity [36] and this improved the quality and safety of care and services [16, 35, 42]. Thus, CP&D was seen as a medium for quality accreditation [38] and improvement [41] to support evidence-based practice [43] and research strategies [44]. Organizational support for nurses' CP&D increased work satisfaction [16, 32, 37], motivation, and retention [39, 41, 45]. Nurses with organizational career development opportunities were engaged in organizations [39] and being rewarded supported their career progress [46]. In addition, clear structures for nurses' career paths supported interprofessional collaboration in organizations [38].

### 3.3. Organizational Structures to Support Nurses' CP&D

The key organizational structures to support nurses' CP&D started from a strategic level in an organization and required a structured framework and continuous evaluation strategies to monitor success (Table 3).

#### 3.3.1. Strategic Level Support for Nurses' CP&D in an Organization

Nurses' CP&D reflected an organization's values, aims and culture, and CP&D needed to be included in the organization's strategy. Future oriented, progressive organizations had a core interest in being committed to nursing staff [37, 43], creating different career opportunities [16] and promoting their career development [43, 46]. In addition, organizations were responsible for promoting the career development of nursing staff [38] and implementing national recommendations and standards to promote their career paths [16, 44, 47].

Recognizing nurses' CP&D was also seen as part of an anticipated strategic policy for workforce and competence needs in an organization, which aimed to match organizational tasks with the competencies needed by the workforce [33]. Matching required considering current staff competences and age structures [48] and reconsidering vacancies [45]. In addition, senior [39] and progressive positions for educated academic staff were needed to engage them in organizations [49]. Matching also required evaluating changes in job descriptions and their effect on workload [38]. In addition, infrastructure for building organizational research capacity was required to support nurses' academic career progression in organizations, including research networks between universities and hospitals [49].

Organizational support for nurses' CP&D required strategic educational partnerships. This referred to planning and executing educational programmes in collaboration with healthcare and learning institutions [31, 38]. Programmes focused on staff re-education [31] and extended their competencies to respond to changing needs [33] and to the development and flexibility of nursing positions and new posts [43]. In addition, strategic educational partnerships included learning institutions that provided lifelong learning possibilities for staff [42].

Supporting nurses' CP&D through organizational strategies also included direct and indirect resourcing and strategic investment. Direct resourcing included budgeting for cumulative salaries according to career progress [46], together with bonuses or other monetary compensation [38, 46]. In addition, supportive, rewarding systems referred to nonmonetary remuneration and included psychological payment for participating in organizational decision-making, progressing in their career [46] and social recognition activities [40]. Direct resourcing also included providing nurses with sufficient equipment to complete their new duties and professional responsibilities [39]. Indirect resourcing for CP&D referred to planning human resources to allow career development and providing sufficient permanent staff and substitutes to enable nurses to participate in education [31, 38, 39, 49]. In addition, vacancies and job description required constant reconsideration and updates so that they corresponded to nurses' career development, increased competencies, special nursing care duties [39], and research and developmental work [49]. In addition, mixed model vacancies between academia and services or public and private sectors supported CP&D [44]. Resourcing also focused on strategic investment for CP&D, as investment in education and competencies improved organizational effectiveness [39] and employees' engagement [49].

#### 3.3.2. Structured Framework for Nurses' CP&D

The structured frameworks to support nurses' CP&D in organizations included nurse manager act as they played a crucial role in career stage mapping and identifying nurses' competencies [38, 50]. These were needed at both unit and individual staff levels [40, 48, 51] to determine nurses' competency levels based on prerequisites set by the organization [38]. At unit levels, updated overviews of available competencies were needed to evaluate how to respond to care needs and anticipate the requirements for future staffing and competencies. At the individual staff level, career stage mapping enabled nurse managers to address suitable responsibilities for staff members with adequate competencies [38, 52]. Career stage mapping consisted of considering the individual nurses' education level, work experience, and duration of work and their competency evaluation [38]. However, identifying nurses' competencies was challenging [49], especially with regards to tacit knowledge [48]. Thus, evaluation required optimal frequency and visibility and tested evaluation criteria tailored to the job description [36].

Standardized qualifications for all career stages were suggested to achieve systematic progress and fair evaluation [36, 44, 51]. Logical career trajectories [41] required clear job descriptions [37, 45] and identifying key knowledge, skills, behaviours, roles, and responsibilities [33] for each career phase. This included definitions for each competency level, from novice to expert [51]. Based on that, all individuals at the same career stage should have developed their skills, capacity, and knowledge to the same standards and have equal responsibilities [33, 52]. The benefits of structured career trajectories were twofold. First, they helped nurses to identify their career and progress opportunities and recognize their knowledge level and further training needs [33]. Second, structured career trajectories served as a tool for nursing managers to evaluate the individual competencies of nurses and nursing staff [16, 51] and to design mentoring and educational programmes for organizations [33]. The standardization and implementation of a structured framework for nurses' career development needed to be evidence based and respond to the purpose of the organization [45]. They should be regularly updated [38] based on committee work in the organization [33] and involve strategic administrators [16] and human resource teams [45] and coordinators [16]. Implementation required nurse managers [33] and nursing staff to be educated about frameworks [53].

Various career opportunities and pathways in organization supported nurses' CP&D [38]. In clinical nursing, these opportunities included daily patient care [39], specialization in some field of nursing [54], and specific tasks in practice [33, 40, 42]. Career opportunities also included more academic options, such as advanced practice [33, 52], project work [39], clinical research [44, 49], and working as a nurse manager [37, 50] or educator [33]. In addition, peer mentoring was considered to be a career opportunity [54].

#### 3.3.3. Evaluating and following up Nurses' CP&D

At an organizational level, constant evaluations and follow ups on implementing nurses' CP&D focused nurses' satisfaction on the existing structures for CP&D [32]. The structures that were evaluated focused on providing education and mentorship and collaboration with educational institutions [38]. In addition, evaluation was focused on outcomes of executed care and service. This referred to the goal of achieving high-quality care and services focused on how nurses could respond to care needs [38] and promote the image of the hospital [38].

### 3.4. Means to Support Nurses' CP&D

The key means to support nurses' CP&D were promoting their competencies, facilitating, and promoting their personal career planning and interpersonal support (Table 3).

#### 3.4.1. Competence Promotion

Career awareness promotion and substance training supported nurses' CP&D. Nurses' career awareness was increased by publishing and presenting career options [49, 53] and success stories [49, 50] in organizations [39]. Career awareness was also promoted by providing nurses opportunities to get acquainted with certain careers, such as developmental work, education, or leadership in practice [35]. Such awareness eased the transfer into academic positions [37]. Substance training referred to nurses' opportunities for lifelong learning [34]. They included formal [37] and tailored educational programmes for staff on their career trajectory [35, 41, 49] and re-education by taking new degrees with the support of an organization [31].

#### 3.4.2. Personal Career Planning

Portfolios [52] and personal career plans were used to support nurses' CP&D. Portfolios were documents, where nurses demonstrated their professional and academic accreditation, competence, and capacity, continuing professional development, and readiness for career progress [52]. Personal career plans or individualized action plans [40, 50] referred to voluntary or compulsory [53] oral discussions [33] or written plans [53]. Career stage frameworks were used as templates for planning [33]. Oral plans involved personal discussions between staff members and nurse managers [45, 48] or with a career coordinator [53] or nurse retentionist. The aim was to identify each nurse's strengths, talents and goals [40], develop, and clarify their individual career prospects [33, 48] and evaluate yearly progress [48] and educational needs [33]. Personal career plans or portfolios were used to evaluate career progress [33, 48, 52].

#### 3.4.3. Interpersonal Support

Interpersonal support for nurses' CP&D referred to support from leaders, mentors, networks, peers, and their work community. Career-oriented work environments [46] referred to organizational values and the crucial role played by nurse managers [31, 37]. These work environments were open and the professional ambitions of the individual nurses were respected [53]. Leadership support by nurse managers played a crucial role in guiding and supporting nurses [38, 40, 45] and career progress [16, 46], in line with organizational developmental policies [38]. The nurse manager's role was to provide opportunities for career progression by engaging nursing staff [39] and enabling them to participate in educational activities [43]. One-to-one career advice and support from the divisional recruitment and retention leads [45] and nurse retentionists [40] were pivotal.

Papers focused on different aspects of mentoring or coaching [50] that supported nurses' CP&D [32, 40]. These were mentoring for evolving career planning [35, 55], identifying different types of career trajectories [32, 35], supporting career transfer phases [40, 50, 54, 56, 57], following up career success [40, 43], and promoting lifelong learning [42]. Nurses' CP&D was mentored by nurse managers [32] and experienced supervisors [54]. The aim of all types of mentoring was to support goal achievement [32, 50], realistic aim setting, and periodical progress assessments [51]. Mentoring was informative [37, 43, 54, 56, 57], emotional, and psychological [37, 55]. Cognitive support [56] and guidance aimed to strengthen nurses' self-efficacy [38, 54], confidence [32, 41], and self-image [41]. Mentoring included discussions about challenges and ethical conflicts [54] that nurses had faced [37, 50]. Mentoring was carried out during regular meetings [35, 56], with reflective discussions [40] and feedback [56], to discover nurses' strengths and developmental needs [40]. Nursing networks and peer support provided interpersonal support for nurses' CP&D. Peers were crucial for sharing knowledge and providing emotional support [37, 40] within informal networks [49].

## 4. Discussion

Organizations recognized nurses' CP&D in relation to the individual's professional development and what the organization needed to do to promote quality services and workforce engagement. There were multiple levels of support for nurses' CP&D. These included the organization's strategic work level, which ensured that resources and purposeful vacancies were available, and the structured framework level, which focused on objective qualification criteria and equal assessments. Different methods were needed to support nurses' CP&D opportunities, including ensuring that they were aware of career planning and providing them interpersonal support. CP&D was linked to an organization's aims. Support was needed within the nursing profession but also through multilayered interprofessional collaboration that was designed and implemented by organizations.

Nurses' CP&D in an organization provided mutual benefits for nurses, employers, and society as the individual and organizational level benefits of nurses' CP&D were strongly interlinked. They increased personal competencies to respond to work challenges and, therefore, the organizational capacity to achieve high-quality focused care and services ([5]). It seemed reasonable for nurses' employers to support their careers, as organizational support for CP&D contributed to the nurses' motivation and their experience of meaningful working life. It also promoted an employer's attractiveness and workforce retention. Despite the importance of nurses' CP&D, there has been a lack of studies, particularly empirical ones.

Nurses have a less clear and narrower career development trajectory than, for example, physicians [48]. Nursing careers often progress from clinical positions to leadership roles or academic tasks, while less attention has been paid to career development in bedside nursing. This is a conspicuous deficiency in nursing and healthcare services, from the perspective of the care quality development and the attractiveness of a clinical career. Patient care is known to be a central reason for choosing nursing as a career [1] and for nurses wanting to develop their careers in this area [39]. Moreover, career development that only focuses on academic and leadership paths is insufficient from a resource point of view, as the need for these roles is minor compared to the need for bedside professionals.

The results of this review emphasize nurses' CP&D in patient care organizations. However, nurses' CP&D is not limited to the boundaries of an organization but extends all the way to the societal need for a highly qualified nursing workforce and equal service provision for clients at a national level. Nurses also need equal treatment wherever they work. That is why national uniform models have been proposed to guide the steps in academic nursing careers [33, 47] and widely promote and standardize equal CP&D possibilities for nurses' in local districts and patient care organizations. These kinds of generic and acknowledged models would also need to cover bedside nursing and play important roles in helping people to choose nursing careers and inform and guide them when they do ([12]). However, generic career models are only part of the story. Regional differences in patient-nurse ratios need to be considered so that campaigns are applicable in districts and organizations. Nurses' trade unions and education play a key role in making CP&D models familiar. Thus, integrating career development competencies and career planning in nursing and nursing leadership education would promote uniformity of nurses' career competencies and awareness.

Nurses' CP&D requires organizational structures and for individuals to play a proactive role. Nurses' organizations need to carry out strategic work on the career information and resources that are crucial. Individuals experience remarkable time and financial pressures during education ([22]). Employers who provide support to overcome these challenges need to implement multifaceted strategic planning and collaboration. These include building financial cooperation models [43] to, at least partly, cover educational expenses. Our study showed that organizational structures needed to focus on identifying equal opportunities to develop competencies and acquire qualifications, as well as provide resources. Clear career paths models and competency assessment criteria contribute to the objectivity and transparency of administrative decisions. However, it should also be noted that professionals who assess how competent personnel are need up-to-date evaluation skills [38] and appropriate competency assessment tools [51].

Even the best organizational structures do not guarantee career development without the individual's own contribution. Every nurse is responsible for their professional development and competencies [38] and plays a key role in their CP&D. Career pursuits require certain readiness [16], namely, awareness of CP&D possibilities, self-efficacy, motivation [38], and emotional readiness [16]. Not least, nurses need to show an appreciation of, and desire to, progress their career [55]. Organizational and individual readiness are known to be interlinked, as organizational support activities have improved nurses' readiness to progress in their careers [16].

Studies on nurses' CP&D had mainly been conducted using empirical qualitative methods, besides which many were theoretical project-based papers. Combining different studies and methods allowed us to map and summarise previous knowledge and provide an overview [24, 25] of nurses' CP&D in healthcare organization. It is noteworthy that the quality of the selected papers varied, particularly within qualitative studies. The strongest areas in them were the congruity between the methodology used and the interpretation of results, as well as data-based conclusions. The weaker areas of reporting concerned philosophical starting points and the researchers' influence on a study. Thus, quality appraisal was needed to increase the transparency of the data. It revealed methodological accuracy to enable reliable and evidence-based development of nurses' CP&D.

### 4.1. Study Strengths and Limitations

The limitations of this study were related to the review process [25], more specifically to literature searches and selection. Because career concepts are widely used in the nursing-related literature and to reach a reasonable number of search results, we had to focus the literature searches on titles of the papers. As a consequence, some relevant papers may have been unintentionally excluded. Also, although the literature selections were carried out based on purposeful criteria and by two researchers, it is possible that some studies dealing with the topic remained unselected. In addition, we only included papers published in English, which may have biased our data.

## 5. Conclusion

Organizations need to see nurses' CP&D as beneficial, necessary for the development of professional nursing practice and a crucial part of the organizational goals needed to achieve high-quality patient care. In contemporary nursing, nurses' CP&D also increasingly contributes to an organization's attractiveness as an employer and to nursing as a career. Clear and transparent structures and models to support and enable nurses' CP&D are central and needed for equal career progression and meaningful working lives. At the same time, nurses' own ambitions and career awareness play crucial roles in CP&D, emphasizing the importance of integrating the topic of CP&D into clinical and leadership education. Further research is needed about the outcomes that nurses' CP&D deliver for their organizations and patient care.

## 6. Implications for Nursing Management

The growing shortage of nursing staff forces their leaders to develop new ways to promote the meaningfulness and attractiveness of nursing, where career opportunities are one central focus. Nurse managers play a key role in ensuring nurses' work engagement [58] and this study helps them to understand nurses' CP&D and its meaning for an employee and organizational benefits and what kind of structures and means are needed to support it. Moreover, the study opens up the key role of nurse managers in supporting nurses' CP&D at an individual level and in nurse communities. Eventually, nurses' CP&D supports the achievement of the overall goal in the organization.

## Figures and Tables

**Table 1:** Description of the studies.

	*n*
*Year*	
2012	1
2013	1
2014	2
2015	2
2016	2
2017	3
2018	4
2019	7
2020	1
2021	4
2022	1
Total	28

*Country*	
UK	9
USA	7
Australia	3
Canada	2
Indonesia	2
Iran	2
Finland	1
Singapore	1
Sweden	1
Total	28

*Method*	
Theoretical	14
Qualitative methods	10
Multiple methods/Delphi	3
Quantitative methods	1
Total	28

*Scope of career in a paper*	
Clinical nursing	16
Clinical academic nursing	8
Nursing leadership	4
Total	28

**Table 2:** Selected studies.

Author(s), year, country	Aim	Methods (scope of career)	Main results or central content
Afriani et al. [16], 2021, Indonesia	To identify the relationship between institutional and nurses' readiness for change in implementing nursing career paths in health centres	Quantitative survey of 93 nurses in public health centres and statistical analyses (clinical nursing)	There was a strong positive relationship between institutional and nurses' readiness. Institutional readiness for change, such as superiors' attitudes and commitment, and nurses' readiness, were needed to implement nursing career paths in public health centres. The nurses considered both had reached a good level

Bramley et al. [35], 2018, UK	To describe the implementation and evaluation results of the bespoke chief nurse fellow programme for frontline junior clinical staff. This was designed to develop skills in innovation, leadership, improvement science, and change management	Theoretical paper: project report (nursing leadership)	The pilot programme had a positive impact on the nurses' professional development and enabled them to become familiar with an alternative career route

Chen and Haller [55], 2015, Canada	To examine the relationship between nurses' career burnout and career wellbeing and how career counsellors can improve their career wellbeing	Theoretical paper (clinical nursing)	Career counsellors can improve nurses' career wellbeing by enhancing effective coping skills and helping goal progress. Combining narrative and social learning career counselling approaches were suggested

Choo et al. [37], 2019, Singapore	To explore the role-transition experiences of assistant nurse clinicians after their first year of appointment	Qualitative primary care study. Interviews with 22 registered nurses and content analysis (clinical nursing)	Previous clinical experience eased the transition. Peer support, mentorship, and training in managerial skills were needed

Cooper et al. [43], 2019, UK	To outline how nursing, midwifery, and allied health professions had been supported to develop clinical academic roles and their contribution to research and innovation in care	Theoretical paper (clinical academic nursing)	Clinical academics needed organizational support to combine clinical and research activity. The hospital's strategic commitment to promoting research and evidence-based practice was important. Clinical staff needed predoctoral opportunities to become familiar with academic work. Work time and mentoring was needed to apply for funding in the postdoctoral phase

Duffield et al. [39], 2014, Australia	To evaluate a career development policy in South Australia, which increased the number of senior staff nurse positions and provided senior registered nurses with time away from clinical duties to undertake agreed projects	Qualitative hospital-based study with 54 senior staff nurses who participated in career structure arrangements and interviews. Analysis method not specified (clinical nursing)	The organization's policy increased the number of senior staff nurse positions and enabled senior RNs to get involved in strategic portfolio projects that aimed to improve organizational effectiveness and care quality. Nurses felt this new policy helped their career and skills and enriched their working lives

Esplen et al. [42], 2018, Canada	To describe a model of education developed based on the novice to expert specialty training framework and its success in offering structured oncology continuing education training to nurses, from undergraduate levels to continued career development in clinical settings	Theoretical paper: project report (clinical nursing)	The paper underlines the importance of nurses' continuing education and certification to ensure high clinical competency. An education institute developed oncology education, including mentorship for lifelong learning and a novice to expert framework with a credit system and a national certification exam. The learning institute partnered with nurse associations. The model notably increased oncology nurses' certifications

Faithfull-Byrne et al. [31], 2017, Australia	To describe a quality improvement project to promote nursing assistants to enrolled nurses	Theoretical paper: project report (clinical nursing)	A hospital and educational institution created a flexible career path for nursing assistants to become enrolled nurses. The two-year study programme included theory and clinical practice. The hospital's nurse educator coordinated the studies and nurse managers helped students to balance their work. Grants covered financial losses due to time away from work. The programme was an effective workforce development strategy for the hospital

Freeman and Gray [33], 2013, UK	To discuss the benefits of a career and development framework for infection prevention and control nurses, developed by a health service organization	Theoretical paper (clinical nursing)	The framework defined responsibilities and professional requirements from practitioner to consultant level, focusing on leadership, learning, evidence, research and development, and clinical practice. It helped individuals to progress their careers, employers, and managers to plan their workforce and mentors to providing support

Jangland et al. [54], 2021, Sweden	To evaluate the implementation of a multifaceted mentoring programme in a large university hospital and describe its value from the perspectives of newly graduated nurses, experienced nurses, and the hospital	Qualitative hospital-based study on 35 nurses, supervisors, and nurse managers, with interviews and thematic analysis (clinical nursing)	Mentors were senior nurses. The mentoring programme comprised research knowledge to guide nursing work, practical and situational guidance for clinical nursing, and group discussions. New nurses found the programme meaningful for their work wellbeing and senior support important in their clinical work. Acting as mentors offered senior nurses a new career opportunity

Jokiniemi et al. [47], 2020, Finland	To formulate, validate, and disseminate policies modelling nurses' career pathway from registered nurse (RN) to advanced practice nurse (APN)	Multiple methods: review, interviews, survey and expert group discussions (clinical academic nursing)	Three competence levels were modelled: RN, specialized nurse, and APN. Central elements in a national RN to APN policy were establishing and enabling new roles, developing education, enhancing appreciation, networking and collaboration, knowledge translation, and governing the roles

Lanada and Forde-Johnston [45], 2021, UK	To reduce variations and standardize job titles, job descriptions, and job plans for clinical nurse educators (CNEs) and identify the academic requirements and professional experience required of each band	Qualitative study with 12 CNEs and 11 senior nurses. Focus group interviews and comparative content analysis (clinical academic nursing)	Job titles, descriptions, and plans were reviewed and CNE roles standardized to reduce variations and inconsistencies. These aligned overall job summaries, teaching activities, and management responsibilities. CNEs were offered career advice and support in line with these career progression descriptions. Staff perceived the new roles as meaningful

Lees-Deutsch et al. [48], 2016, UK	To enable nurses, practitioners, and managers to distinguish between enhancing, expanding, or advancing practice. To clarify points of progression and integrate “advancement” into an acute medical setting	Theoretical paper (clinical nursing)	The paper presents a conceptual framework for nursing career advancement in acute medicine. The framework included five ascending levels of practice and distinguished enhanced, expanded, and advanced ones

Martens et al. [56], 2018, USA	To identify common experiences or barriers during the first year as certified registered nurse anesthetists (CRNAs) moved into management	Qualitative study with 20 members of the American Association of Nurse Anesthesiology	CRNAs had often “fallen” into managerial roles without adequate managerial competencies. Transition support, mentorship, and education were important in the role. People skills were crucial in managerial work
	To identify the knowledge, skills, abilities, and resources needed to ensure a smooth and successful career transition	Interviews and qualitative analysis. (Nursing leadership)	

McGhie-Anderson [46], 2017, USA	To gain an understanding of the social processes associated with the decision of diploma and associate degree nurses to advance academically	Qualitative hospital-based study with 15 diploma and associate degree nurses and 7 other nurses. Interviews and qualitative data analysis (clinical academic nursing)	Rewarding, motivating, and supporting were important factors for nurses' decisions to advance academically. Rewards and positive work environment motivated nurses to progress

Pacho et al. [57], 2023, USA	To present the implementation of a program in a medical centre to support ambulatory care nurses make the transition from direct care to clinical nurse coordinator roles	Theoretical paper: project report (nursing leadership)	The four-week programme combined a web-based toolkit, mentoring network, and shadow shifts. Programme evaluation reflected the nurses' satisfaction with the program and their desired professional advancement

Rahimi et al. [34], 2019, Iran	To investigate the factors affecting career development of nurses in Iran	Delphi study. Hospital-based with 48 nurses and nursing faculty members. Interviews and content analysis, questionnaires, and statistical analyses (clinical nursing)	Central factors affecting nurses' career development were specialization, professional development, and increasing their organizational power and influence

Reville and Foxwell [51], 2017, USA	To present a new competency evaluation tool, the Advanced Practice Palliative Nurse Competency Milestones, to provide a framework for its application and to describe the authors' experience with its use	Theoretical paper (clinical academic nursing)	The tool suggests five competence levels from novice to expert in nine areas of palliative competence

Roddam et al. [49], 2019, UK	To explore the perspectives of aspiring or active clinical academics and health care managers in the nursing, midwifery and allied health professions (NMAHPs) about the benefits, barriers, and enablers of engagement in these career pathways	Qualitative healthcare study focusing on NMAHPs. Workshop data and thematic analysis (clinical academic nursing)	An organizational structure and resources were needed to enable clinical academic career progress, including funding and permanent academic posts. Individuals' research capacity needed to be promoted and networks and mentoring played a central role

Ryley and Middleton [52], 2016, UK	To discuss the implementation of the Welsh Government's advanced practice framework into a Welsh University health board	Theoretical paper (clinical academic nursing)	The paper presents the five stages of the development of the advanced nurse practitioner role

Sandehang et al. [38], 2019, Indonesia	To investigate career mapping for nurses at a new hospital in Jakarta	Qualitative case study in hospital. Discussing CP&D with 8 registered nurses and 6 nurse managers. Content analysis (clinical nursing)	Career mapping aimed to match nurses with adequate skills with optimal work based on prerequisites set by the employer. Mapping focused on the educational level, work experience, and competency assessment. Some challenges were recognized, such as time limitations

Sattler et al. [40], 2021, USA	To describe the innovative nurse retention role implemented in the medical health system in the USA	Theoretical paper: project report (clinical nursing)	A medical health system developed a nurse retention role, including meetings with nurses, building collaborative relationships, revising clinical ladders, cultivating peer mentoring, creating a system-wide recruitment structure, and promoting recognition of effort. Participation in nursing career development increased, nurse turnover dropped significantly and financial savings were achieved

Sheikhi et al. [36], 2015, Iran	To explore nurse leaders' experiences of implementing the nurses' career advancement pathway program in Iran	Qualitative study in hospital with 16 nurse managers. Interviews and content analysis (clinical nursing)	First, shortcomings in performance evaluation were recognized as evaluations were not continuous, there was no agreement between the evaluation criteria and the nurses' job descriptions and evaluation was subjective. Second, they needed to pay attention to the point accumulation so that it did not exceed educational needs. Third, there was an advancement-latitude mismatch. Career advancement may lead to pay rises and higher positions but not role expansion and considerable changes in job descriptions

Smith et al., 2018 [44], Australia	To consider clinician researcher career frameworks. To propose a new pathway, integrating university and health service components to support research career progression within nursing and midwifery practice	Theoretical paper: discussion (clinical academic nursing)	A national researcher career pathway for nurses and midwives was proposed, which comprised six levels from research assistant to chair/clinical professor. Each level specified a title, level of qualification, australian qualifications framework level, role expectations, and examples of role-specific skills

Thompson et al. [50], 2012, USA	To introduce a model emphasizing the importance of mentoring and/or coaching for the aspiring executive nurse leader	Theoretical paper (nursing leadership)	Nurses' career trajectories towards leadership roles can be supported by mentoring and coaching. Mentoring is support, role modelling, discussions, and reflections between a nurse and an experienced executive. Coaching is more orientated to targets and clients and about nurses growing into their new roles. When nurses aspire to executive positions mentoring and coaching can proceed in three phases: basic fundamental knowledge, experiential learning, and advanced system thinking

Tucker et al. [53], 2019, USA	To describe how knowledge translation tools were used to guide the implementation of a professional development and career planning program, developed and piloted in an urban Chicago hospital. The aim was to reduce the turnover of newly hired nurses and looking at how to make this a sustainable change	Theoretical paper: project report and commentary (clinical nursing)	Nurses planned their career with a program coordinator and received information on career developmental possibilities. The Alberta Context Tool collected information on the nurses' work environment, namely, the units' strengths and readiness for change. Programme implementation included assessing, monitoring, and evaluation. Educating and involving nurse managers was central. Nurse turnover decreased. Use of knowledge transition models, context assessment, and expert recommendations for implementing change strategies were important for sustainable change

Wasike et al. [41], 2019, UK	To examine the learning outcomes from a pilot career program developed to advance home care nurses' professional competencies and career planning	Multiple method study with 15 home care nurses. Questionnaires and statistical analyses, interviews, and thematic analysis (clinical nursing)	The six-month program comprised one-day workshops and particularly focused on leadership and quality improvement skills, potential career paths in home care, and conversation confidence. Nurses found the program beneficial for their practice, professional self-image, and planning career

Woolnough and Fielden [32], 2014, UK	To investigate the effects of a career development and mentoring programme on female mental health nurses' career and personal development. The study used a matched comparison group	Qualitative study in mental health nursing with 54 female mental health nurses: 27 who did and 27 who did not receive intervention. Interviews and thematic content analysis (clinical nursing)	The nurses who participated in the program experienced personal development outcomes. Participants progressed more in their career during the 18-month period than the control group

**Table 3:** Nurses' career planning and development (CP&D), organizational structures, and support methods.

Main categories	Subcategories	Groupings
Definitions and rationale for nurses' CP&D	(i) Individual level: target-oriented increase of personal competency to respond to new work challenges	(i) Intentionally directing competencies and content of work
		(ii) Expanding role and professional development
		(iii) Strengthening professional identity and pride
	(ii) Organizational level: resource for improving care and service quality and workforce's commitment	(i) Signalling respect for staff
		(ii) Improving performance
		(iii) Promoting nurses' commitment in organization

Organizational structures to support nurses' CP&D	(i) Nurses' career development and support at the strategic level within an organization	(i) Expressing values and aims in organization
		(ii) Strategic policy for anticipating workforce and competence needs
		(iii) Strategic educational partnerships
		(iv) Resourcing and strategic investment
	(ii) Structured framework for nurses' CP&D	(i) Career stage mapping and identifying nurses' competencies in organization
		(ii) Standardizing qualifications for all career stages
		(iii) Various career opportunities and pathways
	(iii) Evaluation and follow-up of the success of CP&D	(i) Nurses' satisfaction to career planning and development structure
		(ii) Achieving goals in care and service

Means to support nurses' CP&D in organization	(i) Competence promotion	(i) Career awareness promotion
		(ii) Substance training
	(ii) Personal career planning	(i) Portfolios and career plans
		(ii) Follow-up/evaluation
	(iii) Interpersonal support	(i) Career-oriented work environment
		(ii) Leadership support
		(iii) Mentoring
		(iv) Networks and peer support

^*∗*^CP&D = career planning and development.

## Data Availability

The data used to support the findings of this study consist of scientific articles that can be found from electronic databases mentioned in the manuscript.

## Supplementary Materials

Supplementary Materials Supplementary File 1: Prisma-ScR Checklist. Supplementary File 2: the search terms used. Supplementary File 3: quality appraisal of studies.
