# Supplementary material for: Organizational Support for Nurses' Career Planning and Development: A Scoping Review
Source: J Nurs Manag. 2024 Apr 26;2024:8296762. doi: 10.1155/2024/8296762 (PMC11918694; doi:10.1155/2024/8296762)
Supplement: Supplementary Materials — Supplementary File 1: Prisma-ScR Checklist. Supplementary File 2: the search terms used. Supplementary File 3: quality appraisal of studies. [file 8296762.f1.zip › Supplementary file 2.docx]

Supplementary file 2.

The search terms used.

|  | **Search terms and fields** | **Limitations** | **Results** |
| --- | --- | --- | --- |
| CINAHL | nurs* (tit) **AND**  TI "Career development" OR  TI "Career planning" OR TI  "Career adjustment" OR TI "Career acceler*" OR TI  "Career advancement" OR TI "Career continuum" OR TI "Career growth" OR TI "Career mobility" OR TI "Career progression" OR TI "Career trajector*" OR TI "Career transition*" OR TI "Career level*" OR TI "Career stage*" OR TI "Career opportunit*" OR TI "Career option*" OR TI "Career coach*" OR TI "Career counsel*" OR TI "Career educat*" OR TI "Career resource*" OR TI "Career support" OR TI "Career training" OR TI "Career ladder*" OR TI "Career map*" OR TI "Career model*" OR TI "Career path*" OR TI "Career structure*" OR TI "Career system*" OR AB "Career development" OR AB "Career planning" OR AB "Career adjustment" OR AB "Career acceler*" OR AB "Career advancement" OR AB "Career continuum" OR AB "Career growth" OR AB "Career mobility" OR AB "Career progression" OR AB "Career trajector*" OR AB  "Career transition*" OR AB "Career level*" OR AB "Career stage*" OR AB "Career opportunit*" OR AB "Career option*" OR AB "Career coach*" OR AB "Career counsel*" OR AB "Career educat*" OR AB "Career resource*" OR AB "Career support" OR AB "Career training" OR AB "Career ladder*" OR AB "Career map*" OR AB "Career model*" OR AB "Career path*" OR AB "Career structure*" OR AB "Career system*" | - Research article - 2012-2022 - English - Peer reviewed | 355 |
| PubMed | (nurse*[Title] OR "nursing staff"[Title]) **AND**  ("Career development"[Title/Abstract] OR "Career planning"[Title/Abstract] OR "Career adjustment"[Title/Abstract] OR "Career acceler*"[Title/Abstract] OR "Career advancement"[Title/Abstract] OR "Career continuum"[Title/Abstract] OR "Career growth"[Title/Abstract] OR "Career mobility"[Title/Abstract] OR "Career progression"[Title/Abstract] OR "Career trajector*"[Title/Abstract] OR "Career transition*"[Title/Abstract] OR "Career level*"[Title/Abstract] OR "Career stage*"[Title/Abstract] OR "Stage of career"[Title/Abstract] OR "Stages of career"[Title/Abstract] OR "Career opportunit*"[Title/Abstract] OR "Career option*"[Title/Abstract] OR "Career coach*"[Title/Abstract] OR "Career counsel*"[Title/Abstract] OR "Career educat*"[Title/Abstract] OR "Career resource*"[Title/Abstract] OR "Career support"[Title/Abstract] OR "Career training"[Title/Abstract] OR "Career ladder*"[Title/Abstract] OR "Career map*"[Title/Abstract] OR "Career model*"[Title/Abstract] OR "Career path*"[Title/Abstract] OR "Career structure*"[Title/Abstract] OR "Career system*"[Title/Abstract]) | - 2012–2022 - English | 366 |
| Scopus | (TITLE ( nurse*  OR  "nursing staff" )  **AND**  ABS ( "Career development"  OR  "Career planning"  OR  "Career adjustment"  OR  "Career acceler*"  OR  "Career advancement"  OR  "Career continuum"  OR  "Career growth"  OR  "Career mobility"  OR  "Career progression"  OR  "Career trajector*"  OR  "Career transition*"  OR  "Career level*"  OR  "Career stage*"  OR  "Stage* of career"  OR  "Career opportunit*"  OR  "Career option*"  OR  "Career coach*"  OR  "Career counsel*"  OR  "Career educat*"  OR  "Career resource*"  OR  "Career support"  OR  "Career training"  OR  "Career ladder*"  OR  "Career map*"  OR  "Career model*"  OR  "Career path*"  OR  "Career structure*"  OR  "Career system*" ) ) | - 2012-2022 - English - Article - Journal | 363 |
| Web of Science | nurse* OR "nursing staff" (title) **AND**  "Career development" OR "Career planning" OR "Career adjustment" OR "Career acceler*" OR "Career advancement" OR "Career continuum" OR "Career growth" OR "Career mobility" OR "Career progression" OR "Career trajector*" OR "Career transition*" OR "Career level*" OR "Career stage*" OR "Stage* of career" OR "Career opportunit*" OR "Career option*" OR "Career coach*" OR "Career counsel*" OR "Career educat*" OR "Career resource*" OR "Career support" OR "Career training" OR "Career ladder*" OR "Career map*" OR "Career model*" OR "Career path*" OR "Career structure*" OR "Career system*" (topic) | - 2012-2022 - English - Articles | 316 |
| ***In total 1400*** | | | |
