# Supplementary material for: Organizational Support for Nurses' Career Planning and Development: A Scoping Review
Source: J Nurs Manag. 2024 Apr 26;2024:8296762. doi: 10.1155/2024/8296762 (PMC11918694; doi:10.1155/2024/8296762)
Supplement: Supplementary Materials — Supplementary File 1: Prisma-ScR Checklist. Supplementary File 2: the search terms used. Supplementary File 3: quality appraisal of studies. [file 8296762.f1.zip › Supplementary file 3.docx]

Supplementary file 3.

**QUALITY APPRAISAL OF STUDIES**

**Quality appraisal of empirical qualitative studies**

| **Study** | **Evaluation criteria*** | | | | | | | | | | **Scoring by paper** |
| --- | --- | --- | --- | --- | --- | --- | --- | --- | --- | --- | --- |
|  | **#1** | **#2** | **#3** | **#4** | **#5** | **#6** | **#7** | **#8** | **#9** | **#10** |  |
| Choo et al. 2019 | Yes | Yes | Yes | Yes | Yes | Yes | Unclear | Yes | Yes | Yes | 9/10 |
| Duffield et al. 2014 | Unclear | Unclear | Yes | Unclear | Yes | Yes | Unclear | Yes | Yes | Yes | 6/10 |
| Jangland et al. 2021 | Unclear | Unclear | Yes | Yes | Yes | Yes | Yes | Yes | Yes | Yes | 8/10 |
| Lanada et al. 2021 | Unclear | Unclear | Unclear | Unclear | Yes | Unclear | Unclear | Yes | Unclear | Yes | 3/10 |
| Martens et al. 2018 | Yes | Yes | Yes | Unclear | Yes | Yes | Unclear | Yes | Yes | Yes | 8/10 |
| McGhie-Anderson 2017 | Yes | Unclear | Yes | Unclear | Yes | Yes | Yes | No | Yes | Yes | 7/10 |
| Roddam et al. 2019 | Unclear | Yes | Yes | Yes | Yes | Unclear | No | Yes | Yes | Yes | 7/10 |
| Sandehang et al. 2019 | Unclear | Yes | Yes | Yes | Yes | Unclear | Yes | Yes | Yes | Yes | 8/10 |
| Sheikhi et al. 2015 | Unclear | Yes | Yes | Yes | Yes | Unclear | No | Yes | Unclear | Yes | 6/10 |
| Woolnough et al. 2014 | Unclear | Yes | Yes | Yes | Yes | Yes | Unclear | Yes | Yes | NA | 7/10 |
| *In total (out of 10 papers)* | 3 | 6 | 9 | 6 | 10 | 6 | 3 | 9 | 8 | 10 |  |

*NA = Not applicable*

| **Evaluation criteria for qualitative studies* | |
| --- | --- |
| #1 | Is there congruity between the stated philosophical perspective and the research methodology? |
| #2 | Is there congruity between the research methodology and the research question or objectives? |
| #3 | Is there congruity between the research methodology and the methods used to collect data? |
| #4 | Is there congruity between the research methodology and the representation and analysis of data? |
| #5 | Is there congruity between the research methodology and the interpretation of results? |
| #6 | Is there a statement locating the researcher culturally or theoretically? |
| #7 | Is the influence of the researcher on the research, and vice- versa, addressed? |
| #8 | Are participants, and their voices, adequately represented? |
| #9 | Is the research ethical according to current criteria or, for recent studies, and is there evidence of ethical approval by an appropriate body? |
| #10 | Do the conclusions drawn in the research report flow from the analysis, or interpretation, of the data? |

**Quality appraisal of empirical quantitative studies**

| **Study** | **Evaluation criteria*** | | | | | | | |
| --- | --- | --- | --- | --- | --- | --- | --- | --- |
|  | **#1** | **#2** | **#3** | **#4** | **#5** | **#6** | **#7** | **#8** |
| Afriani et al. 2021 | Yes | Yes | NA | NA | NA | NA | Yes | Yes |

*NA = Not applicable*

| **Evaluation criteria for quantitative studies* | |
| --- | --- |
| #1 | Were the criteria for inclusion in the sample clearly defined? |
| #2 | Were the study subjects and the setting described in detail? |
| #3 | Was the exposure measured in a valid and reliable way? |
| #4 | Were objective, standard criteria used for measurement of the condition? |
| #5 | Were confounding factors identified? |
| #6 | Were strategies to deal with confounding factors stated? |
| #7 | Were the outcomes measured in a valid and reliable way? |
| #8 | Was appropriate statistical analysis used? |

**Quality appraisal of empirical multimethod studies**

| **Study** | Elements 1-13 (see below) of the QuADS Criteria  assessed with a scale of 0 (low) to 3 (high) | | | | | | | | | | | | | Scoring by paper |
| --- | --- | --- | --- | --- | --- | --- | --- | --- | --- | --- | --- | --- | --- | --- |
|  | **#1** | **#2** | **#3** | **#4** | **#5** | **#6** | **#7** | **#8** | **#9** | **#10** | **#11** | **#12** | **#13** |  |
| Jokiniemi et al. 2020 | 3 | 3 | 3 | 3 | NA | NA | NA | NA | NA | NA | NA | 2 | 3 | 17/39 |
| Rahimi et al. 2019 | 2 | 3 | 1 | 2 | 0 | 0 | 1 | 1 | 0 | 0 | NA | 0 | 0 | 10/39 |
| Wasike et al. 2019 | 3 | 3 | 3 | 3 | NA | 0 | 3 | 2 | NA | 2 | 0 | 0 | 0 | 19/39 |
| *In total (out of 9 points)* | 8 | 9 | 7 | 8 | 0 | 0 | 4 | 3 | 0 | 2 | 0 | 2 | 3 |  |

*NA = Not applicable*

| **Evaluation criteria for multimethod studies* | |
| --- | --- |
| #1 | Theoretical or conceptual underpinning of the research |
| #2 | Statement of research aim/s |
| #3 | Clear description or research setting and target population |
| #4 | The study design is appropriate to address the stated research aim/s |
| #5 | Appropriate sampling to address the research aim/s |
| #6 | Rationale for choice of data collection tools |
| #7 | The format and content of data collection tool is appropriate to address the stated research aims/s |
| #8 | Description of data collection procedure |
| #9 | Recruitment data provided |
| #10 | Justification for analytic method selected |
| #11 | The method of analysis was appropriate to answer the research aim/s |
| #12 | Evidence that the research stakeholders have been considered in research design or conduct |
| #13 | Strengths and limitations critically discussed |

**Quality appraisal of theoretical studies**

| **Study** | **Evaluation criteria*** | | | | | | **Scoring by paper** |
| --- | --- | --- | --- | --- | --- | --- | --- |
|  | **#1** | **#2** | **#3** | **#4** | **#5** | **#6** |  |
| Bramley et al. 2018 | Yes | Yes | Yes | Yes | Yes | NA | 5/6 |
| Chen & Haller 2015 | Yes | Unclear | Yes | Yes | Yes | Yes | 5/6 |
| Cooper et al. 2019 | Yes | Yes | Yes | Yes | Yes | Yes | 6/6 |
| Esplen et al. 2018 | Yes | Yes | Yes | Yes | Yes | NA | 5/6 |
| Faithfull-Byrne et al. 2017 | Yes | Unclear | Yes | Yes | Yes | Yes | 5/6 |
| Freeman & Gray 2013 | Yes | Unclear | Yes | Yes | Yes | NA | 4/6 |
| Lees-Deutsch et al. 2016 | Yes | Yes | Yes | Yes | Yes | Yes | 6/6 |
| Pacho et al. 2023 | Yes | Yes | Yes | Yes | Yes | Yes | 6/6 |
| Reville & Foxwell 2017 | Yes | Yes | Yes | Yes | Yes | NA | 5/6 |
| Ryley & Middleton 2016 | Yes | Yes | Yes | Yes | Yes | NA | 5/6 |
| Sattler et al. 2021 | Yes | Yes | Yes | Yes | Yes | NA | 5/6 |
| Smith et al. 2018 | Yes | Yes | Yes | Yes | Yes | Yes | 6/6 |
| Thompson et al. 2012 | Yes | Yes | Yes | Yes | Yes | Yes | 6/6 |
| Tucker et al. 2019 | Yes | Yes | Yes | Yes | Yes | Yes | 6/6 |
| *In total (out of 14 papers)* | 14 | 11 | 14 | 14 | 14 | 8 |  |

*NA = Not applicable*

| **Evaluation criteria for theoretical papers* | |
| --- | --- |
| #1 | Is the source of the opinion clearly identified? |
| #2 | Does the source of opinion have standing in the field of expertise? |
| #3 | Are the interests of the relevant population the central focus of the opinion? |
| #4 | Is the stated position the result of an analytical process, and is there logic in the opinion expressed? |
| #5 | Is there reference to the extant literature? |
| #6 | Is any incongruence with the literature/sources logically defended? |
